# The Use of ^13^C-Urea Breath Test for Non-Invasive Diagnosis of *Helicobacter pylori* Infection in Comparison to Endoscopy and Stool Antigen Test

**DOI:** 10.3390/diagnostics10070448

**Published:** 2020-07-03

**Authors:** Hamed Alzoubi, Asma’a Al-Mnayyis, Ibrahim Al rfoa, Amin Aqel, Mohammad Abu-Lubad, Omar Hamdan, Kareem Jaber

**Affiliations:** 1 Department of Microbiology and Immunology, Faculty of Medicine, Mu’tah University, Alkarak 61710, Jordan; aminaqel@hotmail.com (A.A.); abu_lubbad@yahoo.com (M.A.-L.); 2 Department of Clinical Sciences, College of Medicine, Yarmouk University, Shafiq Irshidat St, Irbid 21163, Jordan; asmaa.mnayyis@yu.edu.jo; 3 Gastroenterology Unit, Alkarak Teaching Hospital, Alkarak 61710, Jordan; I.Ibrahim@yahoo.com; 4 Departmrnt of Pathology, Faculty of Medicine, Mu’tah University, Alkarak 61710, Jordan; omarhamdan321988@gmail.com; 5 Deapartment of Pathology, Microbiology and Forensic Medicine, Jordan University, Amman 11924, Jordan; kareemjaber2015@gmail.com

**Keywords:** *Helicobacter pylori*, ^13^C urea breath test, stool antigen test, endoscopy, sensitivity

## Abstract

*Helicobacter pylori* (*H. pylori*) can cause gastritis, peptic ulcer diseases and gastric carcinoma. Endoscopy as the gold standard method of diagnosis is an invasive procedure that might not be suitable in all scenarios. Therefore, this first study in Jordan aimed to assess the non-invasive ^13^C urea breath test (UBT) and stool antigen test for diagnosis of *H. pylori* infection and the successfulness of eradication therapy as alternatives for endoscopy. Hence, a total of 30 patients attending the endoscopy units at Alkarak teaching hospital were asked to complete a questionnaire with demographic and clinical data. They were then tested for *H. pylori* using ^13^C UBT and *H. pylori* stool antigen before having endoscopy. Another 30 patients who were positive for *H. pylori* by endoscopy were tested using both tests 6 weeks post eradication therapy. Results showed that the rate of *H. pylori* detection using endoscopy was 56.7% (17/30). Heartburns (82.3%, *p* value = 0.019), epigastric pain (88.2%, *p* value = 0.007) and vomiting (70.5%, *p* value = 0.02) were the most significant symptoms. Family history of peptic ulcer diseases was significantly associated with an increased risk for having a *H. pylori* positive result (*p* value = 0.02). Compared to endoscopy, the sensitivity of ^13^C UBT for the diagnosis of *H. pylori* was 94.1% (16/17), while it was 76.5% (13/17) for the stool antigen test. The specificity of both tests was equal (76.9%). However, the positive predictive and negative predictive values (84.2% and 90.9%) for ^13^C UBT were higher than those (81.3% and 71.4%) for the stool antigen test. The accuracy of ^13^C UBT was 86.7% compared to 76.7% for the stool antigen test. There was an 87% agreement (20 patients out of 23) between both tests when used to assess success of the eradication therapy. In conclusion, the ^13^C UBT was found to be more sensitive and accurate than the stool antigen test when used for diagnosis; furthermore, it has a comparable outcome to the stool antigen test in assessing the successfulness of the eradication treatment.

## 1. Introduction

*Helicobacter pylori* (*H. pylori*) is a gram-negative, non-spore-forming, spirally shaped bacterium. The bacteria usually colonize the epithelium of the human stomach, in particular the gastric antrum. It is strictly micro-aerophilic and it requires a complex rich growth media in vitro. *H. pylori* is capable of producing a powerful urease which can hydrolyze gastric urea to liberate ammonia and carbon dioxide, neutralizing the gastric acid and increasing the periplasmic pH to alkaline medium, leading to peptic ulcer diseases (PUD) [1,2,3], and is associated with peptic ulcer disease (PUD), gastritis, gastric adenocarcinoma, and type B low-grade mucosal-associated lymphoma [4]. It has been estimated that about 50% of the population is infected with *H. pylori* globally. However, the prevalence, incidence, age distribution and outcomes of infection are significantly different in developed and developing countries [2,5,6].

The majority of *H. pylori*-infected individuals are asymptomatic but around 15% of them develop peptic ulcer disease, and annually around 1–2% of those will experience a major ulcer-related complication. Patients who are infected with *H. pylori* are either diagnosed using non-invasive tests such as the stool antigen, urea breath test (UBT) and serology, or by invasive methods using endoscopic tests including the rapid urease test (RUT), microbiology and histology [3,7].

Growing guidelines currently recommend the use of UBT as a non-invasive test to diagnose *H. pylori* infection and to confirm the eradication of the infection post-treatment [8,9]. Such recommendations are heavily based on studies finding sensitivities of 90–96% and specificities of 88–98% for the UBT as a secondary test, and on the fact that it can be cost effective and more accepted by the patient compared to the invasive gold standard endoscopy [10,11]. It has been suggested that UBT has the highest accuracy compared to other non-invasive tests such as stool antigen testing and serology [12,13]. The UBT is particularly suitable in all clinical conditions where endoscopy is not strictly necessary, and to check the success of eradication regimens [14].

Labelling urea with ^13^C has become increasingly popular because it uses a non-radioactive isotope, therefore the test can be used many times in the same patient and can also be safely performed in children and pregnant women. On the other hand, the urea breath test that uses ^14^C, a technique which is not authorized by most Health Authorities for the diagnosis of *H. pylori*, is associated with a dose of radiation that might be harmful and its use is not permitted for pregnant women or children [15,16].

The main aim of this study was to investigate the sensitivity and specificity of UBT labeled with ^13^C in the diagnosis of *H. pylori*, and in assessing the successfulness of eradication therapy. The results of UBT will be compared to endoscopy as the gold standard invasive test, and to the fecal stool antigen test as a non-invasive test. The study will include 30 patients in each group (a total of 60 patients) from Al karak teaching hospital in Jordan. The findings of the current study are expected to help in assessing the possibility of using UBT as an alternative test to endoscopy in some scenarios, which would be cost-effective and more convenient to the patient.

## 2. Materials and Methods

### 2.1. Study Design and Sample (Patients)

This pilot study was conducted at the endoscopy unit in Alkarak teaching hospital during the period from August 2019 to February 2020. A total of 67 adult patients were randomly recruited for this study. The sample size of this pilot study was calculated as previously described [17]. A written informed consent was obtained from all participants. The study sample was divided into 2 groups as follows: 

The first phase (diagnostic phase) included 37 volunteers, aged between 19 and 63 years, referred for upper GI endoscopy with upper gastrointestinal symptoms. Patients on the appointments list for the Esophagogastroduodenoscope (EGD) procedure were contacted in advance prior to endoscopy. Those who agreed to participate in the study were instructed to avoid any antibiotics, proton pump inhibitors and bismuth for 4 weeks, and instructed to fast at least 4 h prior to the UBT test. Patients were also given a plastic cup and asked to give a stool sample on the same day. A total of 7 patients were excluded because they either did not attend the endoscopy, they did not give a stool sample or have had antibiotics or a proton pump inhibitor recently.

The second group (post-eradication phase) of the study included 30 patients, aged between 19 and 63, who were previously diagnosed with *H. pylori* in the endoscopy unit by the gastroenterologis and prescribed a triple therapy course for 14 days. They were contacted to attend 6 weeks after finishing the course and instructed to fast at least 4 h prior to the UBT test and to give a stool sample on the same day. 

A consent questionnaire was signed by the participants in both phases. The questionnaire included data about age, number of family members smoking, family history of peptic ulcers, previous endoscopies, recent antibiotic or antacid use and symptoms of upper GI such as heartburns, dyspepsia, bloating, vomiting, bad breath smell and epigastric pain. All samples were collected and tested in the endoscopy unit in Alkarak teaching hospital over a 7-month period in 2019–2020. The study was approved by the Ethics Committee at Mutah Faculty of Medicine—Alkarak teaching hospital, (project identification code 201827, date of approval: 23 July 2018).

### 2.2. Endoscopy 

Diagnostic upper GI endoscopy was performed by a gastroenterologist. A total of 3 biopsies were taken after mucosal sampling from the antral region. A rapid urease test was used (HelicotecUT Plus, Strong Biotech Corporation, New Taipei, Taiwan) to detect for the presence of *H. pylori*. Histology was performed as necessary according to local unit guidelines using hematoxylin and eosin stain.

### 2.3. Urea Breath Test

The ^13^C urea breath test was performed two days prior to endoscopy using 75 mg urea (UREA ^13^C breath test Heliforce kit, Beijing Richen-Force Science & Technology Co. Ltd., Beijing, China). The test was performed according to the manufacturer’s instructions, briefly as follows:

A basal breath sample was obtained by asking the patient to take a deep breath then holding it for 10 s before blowing the exhale into a specific bag at zero time. After this, the patient was asked to drink the reagent that contains urea attached to a ^13^C carbon atom in 90 mL of water. Then, 30 min later, the patient was similarly asked to give a breath sample again, which was collected into a new specified bag. The procedure was explained to all patients in advance.

Samples were analyzed by infrared spectrophotometer (IR force-200 Infrared Spectrometer, Beijing Richen-Force Science & Technology Co. Ltd., Beijing, China) to measure the ^13^C isotopic abundance of the 30 min and zero minute breath samples as per the manufacturer’s instructions. The final measured value by the spectrophotometer is called delta over base DOB, and when the DOB value is ≥4.0 ± 0.4, it is considered positive for *H. pylori*. 

### 2.4. Stool Antigen Testing for H. pylori

Stool samples were processed immediately using stool antigen cards (Abon Biopharm hangzhou co. Ltd., Hanghazue, China) according to the manufacturer’s instructions.

### 2.5. Statistical Analysis

Data generated from the study were tabulated as Microsoft Excel sheets (2007) and uploaded to the Statistical Package for Social Sciences (SPSS version 22, IBM Corp., Armonk, NY, USA). The sensitivity, specificity, positive predictive value (PPV) and negative predictive value (NPV) for the 13C-UBT and *H. pylori* stool antigen tests were calculated. Cohen’s kappa test was used to measure inter-observer agreement, Pearson’s chi-squared test and Fisher’s exact for the cells below 5 were used when appropriate. The level of significance was set at a *p*-value of 0.05 to test the hypothesis of no association. *p*-values of <0.05 were considered statistically significant.

## 3. Results

Table 1 shows the data of the 60 patients included in diagnosis and post-eradication phases. Among the 30 patients in the diagnosis phase, there were 13 (43.4%) males, 17 (56.7%) females; the mean patient age was 38 years (19–63 years range) and the mean number of family members was 5.3 (2–9 range). In the post-eradication phase, there were 11 (36.7%) males, 19 (63.3%) females; the mean age was 36.6 (19–63 years range) years and the mean number of family members was 5.9 (2–9 range). 

Table 2 shows the main complaints and risk factors among the 30 patients diagnosed using endoscope. The table shows that heartburn, epigastric pain and vomiting were significantly associated with the presence of *H. pylori* when compared to those who were negative. The *p* values of the significant complaints were 0.019, 0.007 and 0.020, respectively. There was no statistically significant difference for the other complaints such as bloating, dyspepsia and halitosis in both *H. pylori*-positive and -negative results (*p* value < 0.05). The presence of a family history of peptic ulcer diseases was significantly associated with a positive *H. pylori* result (*p* value = 0.02), while gender and smoking were not found to be statistically significant risk factors for having a positive *H. pylori* result (*p* value < 0.05).

**Figure 1:**
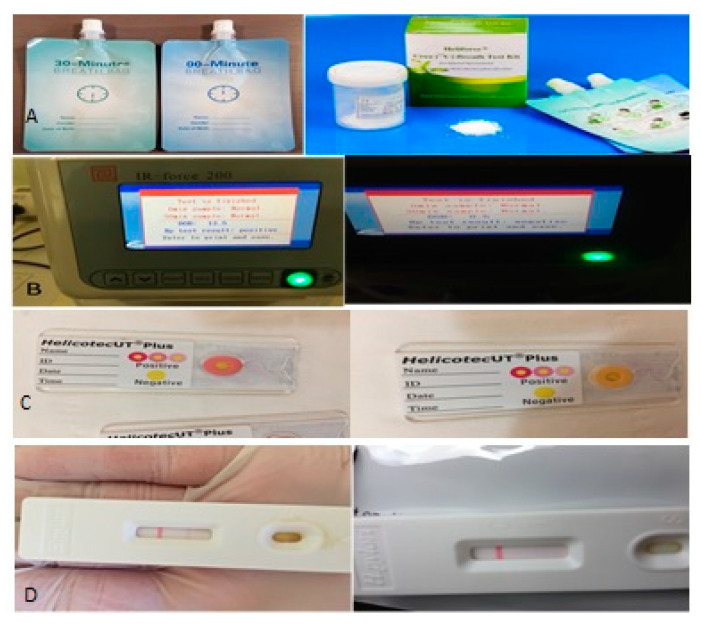
(**A**) The ^13^c breath test kit; (**B**) positive and negative ^13^C urea breath test; (**C**) positive and negative rapid urease test; (**D**) positive and negative stool antigen test.

Figure 1 shows the ^13^C breath test kit and the positive and negative results for ^13^C breath test, rapid urease test and stool antigen test.

**Figure 2:**
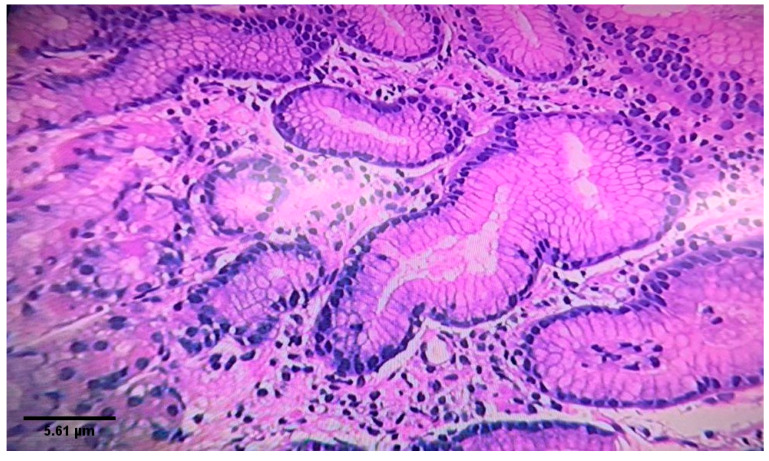
Gastric mucosa showing H. pylori - associated chronic active gastritis. Numerus *H. pylori* bacilli were observed in the lumen of a gastric pit. The lamina propria is infiltrated by mixed inflammatory cell composed mostly of neutrophils and plasma cells. There is no evidence of atrophy, granulomas, intestinal metaplasia, dysplasia and malignancy (Hematoxylin-eosin stain. Magnification ×10).

**Figure 3:**
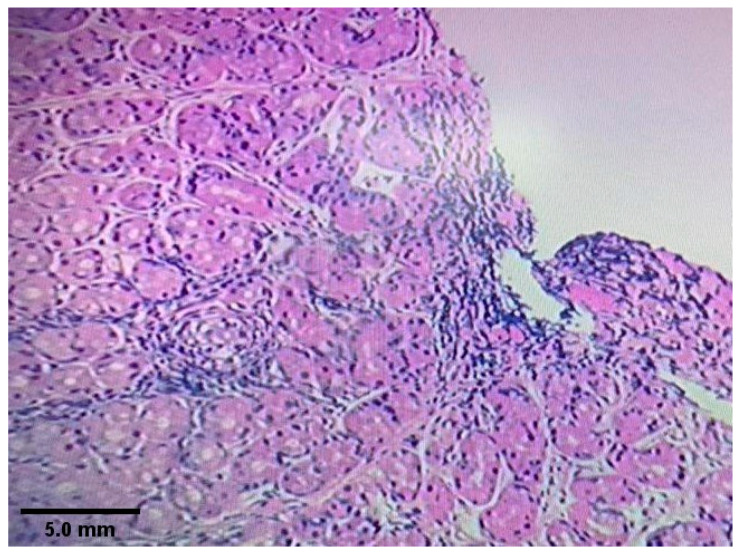
Gastric mucosa showing moderate chronic gastritis with no *H. pylori* microorganisms identified. The lamina propria is infiltrated by chronic inflammatory cell composed mostly of lymphocytes and plasma cells. There is no evidence of *H. pylori* microorganisms, activity, atrophy, granulomas, intestinal metaplasia, dysplasia and malignancy (Hematoxylin-eosin stain. Magnification ×10).

Figure 2 shows histological findings from gastric antrum demonstrating *H. pylori*-associated chronic active gastritis. Figure 3 shows chronic non-specific gastritis with an absence of *H. pylori*. 

Table 3 shows the results of using the ^13^C-urea breath and stool antigen tests for the detection of *H. pylori* infection compared to endoscopy as the gold standard, among 30 patients with variable upper gastrointestinal symptoms. Using the manufacturer-recommended delta over baseline of 4%, the ^13^C-urea breath test had a sensitivity of 16/17 (94.1%) and a specificity of 10/13 (76.9%) compared to a sensitivity of 13/17 (76.5%) and a specificity of 10/13 (76.9%) using the stool antigen test. The false-positive results and false-negative results using the ^13^C-urea breath test were 3/13 (23.1%) and 1/17 (5.9%) compared to 3/13 (23.1%) and 4/17 (23.5%), respectively, using the stool antigen test. The positive predictive value and negative predictive values for the ^13^C-urea breath tests were 84.2% and 90.9%, respectively, while for the stool antigen test, they were 81.3% and 71.4%, respectively. The rate of *H. pylori* according to endoscopy was 17/30 (56.7%).

Table 4 shows the results of using the ^13^C-urea breath and stool antigen tests to assess the successfulness of eradication therapy for 30 patients previously diagnosed to be positive for *H. pylori* infection using endoscopy. The patients received a triple therapy course for 14 days, then, 6 weeks later, the ^13^C-urea breath and stool antigen tests were used to assess eradication. The results indicated that successful eradication was seen in 77% of patients using the *H. pylori* stool antigen test, while it was 67% using the ^13^C urea breath test. The results also showed an 87.0% (20 patients negative by ^13^C UBT and 23 negative by Stool antigen) agreement between the two tests. Both tests revealed that the same seven patients (23.3%) were positive for *H. pylori*. 

Table 5 describes the delta over baseline (DOB) values for 60 patients, who underwent urea breath tests, among the diagnosis and post-eradication groups. According to the manufacturer’s recommendation, a DOB of more than 4% is considered as a positive. At the time of diagnosis, among 30 patients, the mean DOB was 13.1, ranging between 0.1 and 61. Among the 30 patients in the post-eradication group, the mean DOB was 4.6, ranging between 0.1 and 25.6. 

## 4. Discussion

*Helicobacter pylori* is a common human infection affecting around 50% of the world’s population [18]. Infection with *H. pylori* is usually acquired early in life during childhood or adolescence and it is a major cause of gastritis and peptic ulcer diseases. Additionally, it has been associated with an increased risk of gastric cancer [19,20]. The prevalence of *H. pylori* in developing countries is usually higher than in developed countries and can reach more than 80%, which is highly attributed to lower socioeconomic status [21,22]. Other risk factors such as smoking and family history of peptic ulcer diseases were previously suggested to increase the risk of *H. pylori* infection [23,24].

Several noninvasive (stool antigen test, urea breath test, and serology) tests and invasive (endoscopy coupled with histology, rapid urease test, and microbiological culture) tests are being used to detect *H. pylori* infection [25,26]. Endoscopic procedures are currently considered the gold standard test to assess patients with peptic ulcer disease and the presence of *H. pylori*. However, endoscopy can be of more risk than non-invasive tests, and might be uncomfortable for many patients. In addition, it is more expensive compared to non-invasive tests [14]. Therefore, there is an ongoing search for an accurate test that can be easily administered and can be used in different scenarios conveniently, such as in pregnant women and children, and when endoscopy is not applicable. Non-invasive tests, such as the urea breath test and stool antigen test, were suggested to be more convenient to the patient and cheaper when compared to endoscopy [14,25]. In this study, we made assessments using the urea breath test and stool antigen test to diagnose *H. pylori* infection and the successfulness of eradication therapy in patients who underwent endoscopy as a gold standard reference point.

In the current study, the detection rate of *H. pylori* among the 30 patients in the diagnostic phase using endoscopy was 56.7%. This rate is in agreement with what was previously found in Jordanian adults, where the prevalence of infection with *H. pylori* was found to be between 56% and 82% among adults [27,28]. However, our 56.7% rate was much higher than that found in children aged 4–18 years in a study carried out by Altamimi et al., 2019 [29] using the ^13^C urea breath test. Differences in the study population, ages and other risk factors are expected to affect the rate between different studies. In addition, it can be concluded that infection with *H. pylori* can also be acquired beyond the childhood period, based on different prevalence found in these studies among children and adults.

Patients who attended the endoscopy unit were complaining of different gastrointestinal symptoms such as heartburn, bloating, epigastric pain, vomiting, bloating and bad breath smell. However, the only statistically significant symptoms that were found to be associated with positive *H. pylori* detection were heartburn, epigastric pain and vomiting, which were previously suggested to be among the significant presenting symptoms of *H. pylori* infection [30]. The presence of other non-statistically significant symptoms can be related to many factors, such as study sample size, presence of other underlying gastrointestinal disorders and not fully remembering all symptoms by the patient. Among the studied risk factors, family history of peptic ulcer disease was found to significantly increase the risk of having *H. pylori* infection. Other factors, such as gender and smoking, were not found to cause a significant increase in the risk of having *H. pylori.*

Jordan is one of the countries where smoking is prevalent, and it is estimated that around 33% of the population are current smokers [31]. In the current study, which was not a case-control study, around half of the participants in the diagnostic phase, in those found positive and negative for *H. pylori*, were smokers, and this can explain why smoking was not found as a significant risk factor for having *H. pylori*. However, smoking has been previously suggested to adversely affect peptic ulcers, making them difficult to treat. Smoking is thought to adversely affect the immune system, causing damage and impairment of normal gastroduodenal defense mechanisms against the organism, and causes a reduction in local gastrointestinal antioxidants [32].

The use of the ^13^C urea breath test and *H. pylori* stool antigen in this study aimed to assess their sensitivity and specificity in diagnosing *H. pylori* compared to the gold standard endoscopy. Additionally, they were used to assess the successfulness of *H. pylori* eradication after using the appropriate regime by the gastroenterologists. Our results showed that the ^13^C urea breath test performed better than the *H. pylori* stool antigen for diagnosis. The ^13^C urea breath test was more sensitive and accurate than *H. pylori* stool antigen testing. The sensitivity and accuracy of the ^13^C urea breath test was 94.1% and 86.7%, compared to 76.5% and 76.7% using the *H. pylori* stool antigen test, respectively. However, the specificity of the ^13^C urea breath test and *H. pylori* stool antigen testing was equal and was 76.9%. The current results showed that the sensitivity of the ^13^C urea breath test is within what was previously described and is much better than the stool antigen test, but the specificity of both tests was less than what was previously found by other studies, which mentioned a sensitivity of the urea breath test of more than 90% with a specificity of more than 88%, the same as for *H. pylori* stool antigen testing, but with a specificity of 78% [10,11,12,13].

Different factors have been suggested to improve urea breath test sensitivity. Such factors include fasting overnight instead of 4 h, avoiding some foods, setting cut-offs for delta over baseline between 2% and 5% which showed equal accuracy [33]. Additionally, increasing sample size in future studies is highly recommended. The false negative results can be suggested to be due to low colonization of *H. pylori* in the stomach, which leads to low *H. pylori* urease levels or low load in the stool, which are undetectable by the tests, in addition to specific collection and storage requirements, which are a common problem for many faecal tests and could affect the results. Similarly, dead bacteria or coccoid forms of *H. pylori* can be the reason for having false positive results in the stool antigen test.

Eradication of *H. pylori* remains a challenge, and there are continual efforts to identify factors that predict treatment success. Therefore, the urea breath test and *H. pylori* stool antigen testing were used in the current study to assess the status post-H. *pylori* eradication 6 weeks after finishing the triple therapy.

Successful eradication was seen in around 77% of patients using *H. pylori* stool antigen testing, while it was around 67% using the urea breath test. Though it was lesser in the urea breath test than *H. pylori* stool antigen testing, statistical analysis showed an 87% agreement (20 patients out of 23) between both tests. Failure of eradication can be explained by many factors such as incompliance with treatment, presence of risk factors such as heavy smoking and probably the presence of resistant strains of *H. pylori*, which requires further studies. Overall, with the high level of agreement between both tests, the non-invasive tests can still be suggested as a tool for the screening of the successfulness of eradication treatment combined with the assessment of clinical symptoms since both tests are cheap and easy to administer, and more comfortable than gold standard endoscopy.

## 5. Conclusions

The current pilot study showed a rate of *H. pylori* of 56.7% among the studied population. In addition, heartburn, epigastric pain and vomiting were significant symptoms that were found to be associated with the presence of *H. pylori* in the study sample. The ^13^C UBT was found to be more sensitive and accurate than the stool antigen test when used for diagnosis. The sensitivity of the ^13^C UBT for the diagnosis of *H. pylori* was 94.1% and was 76.5% for stool antigen test. Both tests showed a comparable outcome in assessing the successfulness of the eradication treatment. Therefore, the ^13^C UBT can be used as a noninvasive test for the diagnosis of *H. pylori* with clinical correlation, and it can be equally used as fecal antigen test to assess the success of the eradication treatment. 

## Figures and Tables

**Table 1:** Data of the study population in diagnosis and post-eradication phases.

Characteristic	Diagnosis Phase	Post-Eradication Phase
**Gender**		
Male	13 (43.4%)	11(36.7%)
Female	17 (56.7%)	19 (63.3%)
**Age**		
Mean ± SD	38.0 ± 12.0	36.6 ± 12.0
Range	19–63	19–63
**Family members**		
Mean ± SD	5.3 ± 1.9	5.9 ± 1.5
range	2–9	2–9

**Table 2:** Main complaints and risk factors among patients diagnosed using endoscopy.

Symptom Yes No	*H. pylori* + ve	*H. pylori* − ve	*p*-Value
Heartburn	14 3	5 8	0.019
Bloating	8 9	10 3	0.11
Epigastric pain	15 2	5 8	0.007
Dyspepsia	8 9	9 4	0.25
Vomiting	12 5	3 10	0.02
Halitosis	10 7	6 7	0.51
**Risk factors**			
Male Female	10 7	9 4	0.6
Smoking	8 9	8 5	0.46
Family history of PUD	14 3	5 8	0.02

PUD, Peptic ulcer disease.

**Table 3:** Results using the ^13^C-urea breath and stool antigen tests for the detection of *h. pylori* compared to endoscopy.

Test	Gold Standard Endoscopy		Total	PPV/NPV/Accuracy %
**^13^c-urea breath**	**Positive**	**Negative**		
Positive	16 (94.1%)	3 (23.1%)	18 (60.0%)	84.2/90.9/86.7
Negative	1 (5.9%)	10 (76.9%)	12 (40.0%)	
Total	17 (56.7%)	13 (43.3%)	30	
**Stool Ag**	**Positive**	**Negativ** **e**		
Positive	13 (76.5%)	3 (23.1%)	16 (53.3%)	81.3/71.4/76.7
Negative	4 (23.5%)	10 (76.9%)	14 (46.7%)	
Total	17 (56.7%)	13 (43.3%)	30	

PPV: positive predictive value. NPV: Negative predictive value.

**Table 4:** Results of the ^13^C-urea breath and stool antigen tests in the post-eradication group.

Test	Stool Ag Test		Total
**Urea Breath**	Positive	Negative	
Positive	7 (100%)	3 (13%)	10 (33.3%)
Negative	0 (0.0%)	20 (87.0%)	20 (66.7%)
Total	7 (23.3%)	23 (76.7%)	

**Table 5:** Delta over baseline (DOB) for patients in diagnosis and post-eradication groups.

DOB	Group One N = 30	Percentage	Group Two N = 30	Percentage
0.1–3.9	14	46.7%	20	66.7%
4–9.9	4	13.3%	5	16.7%
10–19.9	6	20%	3	10
20–29.9	2	6.7%	2	6.7%
30–39.9	2	6.7%	0	0.0%
40–49.9	0	0.0	0	0.0%
50–59.9	1	3.3%	0	0.0
60–69.9	1	3.3%	0	0.0%
Mean ± SD	13.1 ± 16.1			4.6 ± 6.7
Range	0.1–61			0.1–25.6
